# The Clinical and Economic Consequences of Delayed Transcatheter Aortic Valve Replacement

**DOI:** 10.1016/j.shj.2025.100742

**Published:** 2025-10-25

**Authors:** Sreekanth Vemulapalli, Mark Russo, Shannon Murphy, Soumya Chikermane, Seth Clancy, Curtiss Stinis

**Affiliations:** a Division of Cardiology, Department of Medicine, Duke University Medical Center, Durham, North Carolina, USA; b Division of Cardiac Surgery, Rutgers-Robert Wood Johnson Medical School, New Brunswick, New Jersey, USA; c Global Health Economics and Reimbursement, Edwards Lifesciences, Irvine, California, USA; d Division of Interventional Cardiology, Scripps Clinic and Research Foundation, La Jolla, California, USA

## Abstract

**Background:**

Delays in access to aortic valve replacement, the recommended treatment for aortic stenosis (AS), is common; however, the impact of this delay is not clearly known. This study aims to examine the clinical and economic consequences of delayed transcatheter aortic valve replacement (TAVR) in patients with clinically significant AS.

**Methods:**

We analyzed 4069 patients with clinically significant AS who underwent TAVR between July 2019 and June 2023 using the Optum Market Clarity database. Patients were categorized as timely TAVR (≤90 days from diagnosis) or delayed TAVR (>90 days or urgent/emergent procedure). Clinical outcomes included all-cause mortality, heart failure hospitalizations, stroke, and composite endpoints over 3 years. Economic outcomes included total health care costs and hospitalization utilization. Multivariable Cox proportional hazards and generalized linear models were used for analysis.

**Results:**

Of 4069 patients, 2051 (50.4%) received timely TAVR and 2018 (49.6%) received delayed TAVR. Delayed patients had higher frailty scores (10.9 vs. 8.5; *p* < 0.01) but similar comorbidity burden. At 3 years, delayed TAVR was associated with significantly higher composite outcomes (50.1 vs. 36.8%; hazard ratio (HR): 1.52; *p* < 0.01), mortality (19.5 vs. 13.7%; HR: 1.50; *p* < 0.01), and heart failure hospitalizations (38.4 vs. 26.5%; HR: 1.59; *p* < 0.01). Disabling stroke was not statistically significant (10.0 vs. 7.9%; HR: 1.25; *p* = 0.0558). Delayed patients incurred $36,740 higher health care costs over 3 years ($182,470 vs. $145,730; *p* < 0.01), driven primarily by increased hospitalizations ($22,127 difference). Results remained significant when restricted to elective procedures only.

**Conclusions:**

Delayed TAVR is associated with substantial clinical and economic consequences, including a 50% higher mortality risk and $36,740 in excess costs over 3 years. These findings support the importance of timely intervention and health care system investments to reduce TAVR wait times.

## Highlights

• Delayed TAVR beyond 90 days increases 3-year mortality risk by 50% (HR: 1.50).
• Delayed treatment results in $36,740 excess health care costs over 3 years.
• Heart failure hospitalizations are 59% more likely with delayed TAVR (HR: 1.59).
• Adverse outcomes persist even when restricting analysis to elective procedures.
• Nearly half of patients experience treatment delays beyond recommended benchmarks.

## Introduction

Despite the established efficacy of transcatheter aortic valve replacement (TAVR) for prompt intervention in symptomatic severe aortic stenosis (AS),^1^ and most recently in asymptomatic severe AS,^2^^,^^3^ significant delays in treatment remain commonplace. The exponential growth in TAVR demand has resulted in current capacity becoming overwhelmed, with inadequate access to TAVR being observed in some jurisdictions.^4^^,^^5^ While studies have documented geographic and socioeconomic barriers to TAVR access and health care costs associated with delays in the United States, comprehensive analyses of the long-term clinical and economic consequences of treatment delays remain limited. This imbalance has translated to prolonged wait times and increasing morbidity and mortality on the wait-list.^4^ Population-based studies have documented substantial variation in access to TAVR, with median wait times exceeding recommended benchmarks and significant proportions of patients waiting 6 months or longer for intervention.^6^^,^^7^

Current evidence gaps exist regarding the comprehensive clinical and economic impact of delayed TAVR in real-world populations, particularly in understanding how delays affect mortality, hospitalizations, and health care costs in an integrated analysis. While previous studies have examined either clinical outcomes or health care costs separately, few have provided comprehensive assessments of both clinical and economic consequences of delayed TAVR with robust adjustment for patient complexity and comorbidities.

As such, we conducted an analysis using a large, real-world data set to examine both the clinical and economic consequences of delayed TAVR in patients with clinically significant AS. Our objectives were to: 1) quantify the clinical impact of delayed TAVR on mortality, hospitalizations, and major cardiovascular events; 2) assess the economic burden associated with treatment delays; and 3) characterize the relationship between delay duration and health care resource utilization patterns. We hypothesized that delays in TAVR beyond 90 days from clinically significant AS diagnosis would be associated with significantly worse clinical outcomes and higher health care costs, with effects that persist even after successful valve replacement.

## Methods

### Data Source

This research used Optum’s deidentified Market Clarity data (2007 through 2024Q3), an extensive health care database that integrates data from both Optum-affiliated and nonaffiliated providers in the United States. The database is derived from longitudinal inpatient and outpatient electronic health records (EHRs) for 81 million US patients across all 50 states who receive care from one of the participating provider organizations. Additionally, a subset of these patients has linked medical and pharmacy claims data. The combined electronic health records and claims data provide patient demographics, medical details (such as lab test results), procedures, outcomes, and medications, as well as provider information and notes. Natural language processing is applied to unstructured clinical notes, enabling extraction of detailed clinical information, including diagnosis codes, symptoms, biomarkers, and laboratory values.^8^ Mortality in this database is ascertained through linkage to multiple data sources, including the Social Security Administration Death Master File, Centers for Medicare and Medicaid Services records, facility discharge records, health plan enrollment data, EHRs, and obituary databases.

For cardiac patients, the database captures detailed clinical parameters including echocardiographic findings, relevant biomarkers, and comprehensive documentation of cardiovascular symptoms and complications. The integrated nature of the data set allows tracking of both clinical outcomes and health care resource utilization.

All data were deidentified and accessed in compliance with the Health Insurance Portability and Accountability Act. As a retrospective analysis of a deidentified database, the research was exempt from Institutional Review Board review under 45 Code of Federal Regulations 46.101(b) (4).

### Study Population

We identified adult patients (≥18 years) with clinically significant AS who underwent TAVR between July 2019 and June 2023. Clinically significant AS was defined as symptomatic AS with either a heart failure (HF) diagnosis or documentation of at least 2 symptom notes within ± 30 days of AS diagnosis. Qualifying symptoms included chest pressure, dyspnea on exertion, dyspnea, syncope/presyncope, or fatigue. The clinically significant AS date was the later of either the first AS diagnosis date or the first symptomatic date.

Inclusion criteria required that a patient’s TAVR procedure occurred within 2 years of their clinically significant AS diagnosis, they had continuous health plan enrollment with medical and pharmacy coverage from 6 months before AS diagnosis through valve replacement, and echocardiographic documentation within 30 days of AS diagnosis. Patients were excluded if they were under 18 years of age or had a prior aortic valve replacement before their AS diagnosis.

Patients were categorized into 2 groups based on treatment timing: timely TAVR (elective procedure within 90 days) versus delayed TAVR (urgent/emergent procedure or >90 days after diagnosis). Urgent/emergent procedures were identified based on hospital admission status as provided in the Optum Market Clarity database, or if missing, based on documentation of emergency department services. We chose 90 days as a clinically meaningful threshold that allows time for appropriate evaluation while identifying significant delays. This threshold aligns with established quality metrics, including the 90-day target recommended by the American Heart Association's Target-aortic stenosis registry^9^ and the performance measure for undergoing aortic valve replacement after diagnosis of severe, symptomatic AS established in the 2024 American Heart Association/American College of Cardiology Clinical Performance and Quality Measures.^10^

### Variables of Interest

All time-to-event clinical outcomes were measured over 3 years following the TAVR procedure date. Primary endpoints included time to all-cause death, time to HF hospitalization, time to disabling stroke hospitalization (defined as hospitalization where ischemic or hemorrhagic stroke is the principal diagnosis), and time to composite endpoint (death, HF hospitalization, or disabling stroke hospitalization).

Economic endpoints were assessed over 3 years following TAVR discharge and included total number of admissions per year (all cause, cardiovascular, and HF-specific) and mean health care costs per year (total and inpatient costs). All costs were annualized based on days enrolled during each year, with enrollment requiring commercial, Medicare, Medicaid, or other health plan coverage with both medical and pharmacy benefits.

Patient demographics included age, sex, race (Caucasian, other), geographic region (Midwest, Northeast, South, West), and payer type (commercial, Medicare, other). Clinical characteristics captured in the year prior to TAVR admission included comorbidity burden assessed using the Elixhauser Comorbidity Index,^11^ a well-established measure that quantifies patient morbidity based on 31 diagnostic categories derived from the International Classification of Diseases codes, with higher values reflecting greater comorbidity burden. Frailty was measured using the Hospital Frailty Risk Score,^12^ a validated tool that uses administrative data to identify patients at elevated risk for adverse outcomes, with scores above 5 representing intermediate-to-high frailty risk. Additional clinical variables included the presence of a bicuspid aortic valve and procedure characteristics, including procedure year and elective status.

### Statistical Analyses

Baseline patient characteristics were summarized using descriptive statistics. Continuous variables were reported as means with standard deviations, and categorical variables were presented as frequencies with percentages. Baseline characteristics were compared between timely and delayed TAVR groups.

Clinical outcomes were analyzed using survival analysis methods based on all patients, regardless of survival status after the TAVR procedure. Adjusted analyses utilized multivariable Cox proportional hazards models for death and composite outcomes, with event rates reported as 1 minus survival probability. For HF hospitalizations and stroke, multivariable Fine-Gray subdistribution hazards models were employed with death as a competing risk, with results presented using cumulative incidence functions. The proportional hazards assumption was tested using the Supremum test in Statistical Analysis Software (SAS), v 9.4 (Cary, North Carolina).

Economic outcomes were analyzed for patients who survived TAVR and remained enrolled in their health plan for at least 1 day following discharge. Hospitalization rates and health care costs were compared between timely and delayed TAVR groups using generalized linear models, with gamma distribution for costs and Poisson distribution for admission counts, both employing log link functions. Analyses were conducted separately for each post-TAVR year, including only patients with enrollment during that year.

All models were adjusted for demographic and clinical variables, including age, sex, Caucasian race, region, payer, procedure year, Elixhauser score, Hospital Frailty Risk Score, and presence of bicuspid aortic valve. A sensitivity analysis was performed restricting the cohort to elective TAVR procedures only to assess the robustness of findings when excluding urgent and emergent cases. All analyses were performed using SAS.

## Results

**Figure 1:**
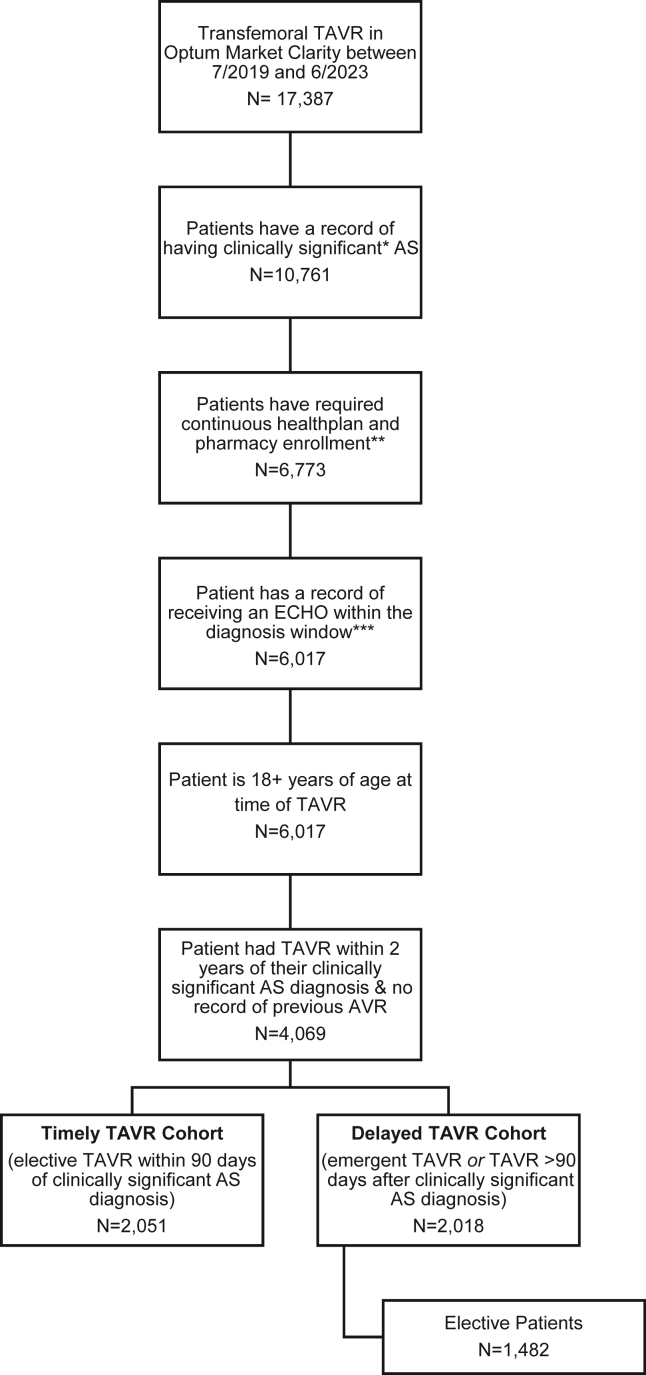
**Attrition diagram.** ∗ Clinically significant (CS) defined as symptomatic AS (HF diagnosis or at least 2 symptom notes) with an AS diagnosis within ± 30 days of symptoms. Symptoms include the following categories—*Chest pressure*: angina pectoris, exertional angina, resting angina, chest pressure, precordial chest pain; *DOE*: doe, soboe, dyspnea on effort; *Dyspnea*: shortness of breath, dyspnea, paroxysmal nocturnal dyspnea, labored breathing, breathlessness, paroxysmal dyspnea; *Syncope/Presyncope*: syncope, syncope and collapse, exertional syncope, fainting, fainting spell, presyncope; and *Fatigue*: chronic, severe, and/or exertional fatigue. ∗∗ Continuous health plan enrollment (i.e., commercial, Medicare Advantage, Medicaid, other) with medical and pharmacy coverage in the 6 months before the clinically significant AS date through AVR. ∗∗∗ Must have a record for an echo in the 30 days before the first AS diagnosis through the clinically significant AS date Abbreviations: AS, aortic stenosis; AVR, aortic valve replacement; ECHO, echocardiogram; HF, heart failure; TAVR, transcatheter aortic valve replacement.

A total of 10,761 patients with clinically significant AS were identified in the Optum Market Clarity data set from 2007 to 2024Q3. After applying inclusion and exclusion criteria, 4069 patients met all study requirements and comprised the final analytic cohort (Figure 1). The most common reasons for exclusion were lack of continuous health plan enrollment (n = 3988) and absence of TAVR within 2 years of clinically significant AS diagnosis (n = 1948).

Of the 4069 patients in the final cohort, 2051 (50.4%) received timely TAVR (within 90 days of clinically significant AS diagnosis) and 2018 (49.6%) received delayed TAVR (1650 received TAVR after 90 days and 368 received urgent/emergent TAVR within 90 days). Nearly all patients (99.5%, n = 4047) survived TAVR and had at least 1 day of health plan enrollment during the 30-day postdischarge period, making them eligible for follow-up analyses. Among the delayed TAVR group, 536 patients (26.6%) underwent urgent or emergent procedures, while 1482 (73.4%) had elective procedures performed more than 90 days after diagnosis.

Baseline patient characteristics are presented in Table 1. The cohorts were well-balanced across most demographic and clinical variables. The mean age was similar between groups (76.5 ± 7.9 years for timely TAVR vs. 75.6 ± 8.5 years for delayed TAVR), as were sex distribution (42.5 vs. 41.4% female) and racial composition (92.0 vs. 89.6% Caucasian). Notable differences emerged in clinical risk profiles. Patients in the delayed TAVR group had significantly higher Hospital Frailty Risk Scores compared to the timely group (10.9 ± 9.4 vs. 8.5 ± 7.7, representing a 28% higher frailty burden). Elixhauser Comorbidity Index scores were similar between groups (6.6 ± 2.8 vs. 6.6 ± 2.7). Regional variation was observed, with the Midwest and Northeast having higher proportions of delayed TAVR cases, while the South had more timely procedures. The delayed group also had a slightly lower prevalence of bicuspid aortic valve disease (4.3 vs. 5.3%). Finally, the distribution of timely versus delayed TAVR remained relatively consistent across procedure years, with delayed TAVR proportions ranging from 45.5 to 54.2% (Appendix A), and no substantial increase observed during the COVID-19 pandemic years of 2020-2021.

**Table 1:** Patient characteristics at the time of clinically significant AS diagnosis

Cohort	Timely TAVR	Delayed TAVR	Total
Sample size	2051 (50.4% of Total)	2018 (49.6% of Total)	4069 (100%)
Alive and enrolled in follow-up period after TAVR discharge	2046 (99.8%)	2001 (99.2%)	4047 (99.5%)
Age (in y)	76.5 (7.9)	75.6 (8.5)	76.1 (8.2)
Female	872 (42.5%)	835 (41.4%)	1707 (42%)
Caucasian race/ethnicity	1886 (92%)	1808 (89.6%)	3694 (90.8%)
Payer			
Commercial	315 (15.4%)	308 (15.3%)	623 (15.3%)
Medicare	1699 (82.8%)	1660 (82.3%)	3359 (82.6%)
Other	37 (1.8%)	50 (2.5%)	87 (2.1%)
Region			
Midwest	871 (42.5%)	922 (45.7%)	1793 (44.1%)
Northeast	379 (18.5%)	423 (21%)	802 (19.7%)
South	528 (25.7%)	409 (20.3%)	937 (23%)
West	273 (13.3%)	264 (13.1%)	537 (13.2%)
AVR y			
2019 (July–December)	248 (12.1%)	294 (14.6%)	542 (13.3%)
2020	494 (24.1%)	505 (25%)	999 (24.6%)
2021	532 (25.9%)	518 (25.7%)	1050 (25.8%)
2022	537 (26.2%)	449 (22.2%)	986 (24.2%)
2023 (January–June)	240 (11.7%)	252 (12.5%)	492 (12.1%)
Elixhauser score	6.6 (2.7)	6.6 (2.8)	6.6 (2.8)
Hospital Frailty Risk Score	8.5 (7.7)	10.9 (9.4)	9.7 (8.7)
Bicuspid aortic valve	108 (5.3%)	86 (4.3%)	194 (4.8%)

AS, aortic stenosis; AVR, aortic valve replacement; TAVR, transcatheter aortic valve replacement.

### Clinical Outcomes

**Figure 2:**
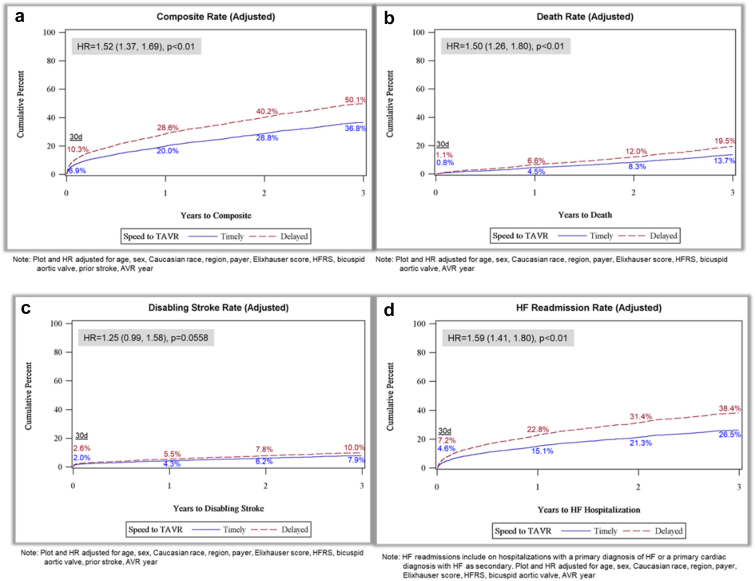
Adjusted risk of composite endpoint (death, disabling stroke, or heart failure readmission) and each component at 3 years for: (a) composite endpoint and its components, (b) all-cause death, (c) disabling stroke, and (d) HF readmission. Abbreviations: AVR, aortic valve replacement; HF, heart failure; HFRS, hospital frailty risk score; HR, hazard ratio; TAVR, transcatheter aortic valve replacement.

Delayed TAVR was associated with significantly worse clinical outcomes across most measured endpoints over the 3-year follow-up period (Figure 2). The composite outcome of death, HF hospitalization, or disabling stroke hospitalization occurred in 50.1% of delayed TAVR patients compared to 36.8% of timely TAVR patients at 3 years, representing a 52% increased risk (hazard ratio [HR]: 1.52; 95% confidence interval [CI]: 1.37-1.69; *p* < 0.01), adjusted for age, sex, race, region, payer, procedure year, comorbidity burden, frailty, and bicuspid aortic valve) (Figure 2A). The survival curves separated early and continued to diverge throughout the follow-up period, with an absolute difference of 13.3% at 3 years.

All-cause mortality was significantly higher in the delayed group, with 3-year mortality rates of 19.5 versus 13.7% in the timely group (HR: 1.50; 95% CI: 1.26-1.80; *p* < 0.01) (Figure 2B). This represented a 50% increased risk of death associated with delayed TAVR, with an absolute mortality difference of 5.8% at 3 years.

HF hospitalizations showed the most pronounced difference between groups. At 3 years, 38.4% of delayed TAVR patients experienced HF readmissions compared to 26.5% of timely patients (HR: 1.59; 95% CI: 1.41-1.80; *p* < 0.01) (Figure 2D). This represented nearly a 12% absolute difference in HF hospitalization rates.

Disabling stroke hospitalization rates were higher in the delayed group, with a 3-year incidence of 10.0 versus 7.9% in the timely group (HR: 1.25; 95% CI: 0.99-1.58; *p* = 0.0558) (Figure 2C), though this difference was not statistically significant.

### Economic Outcomes

**Figure 3:**
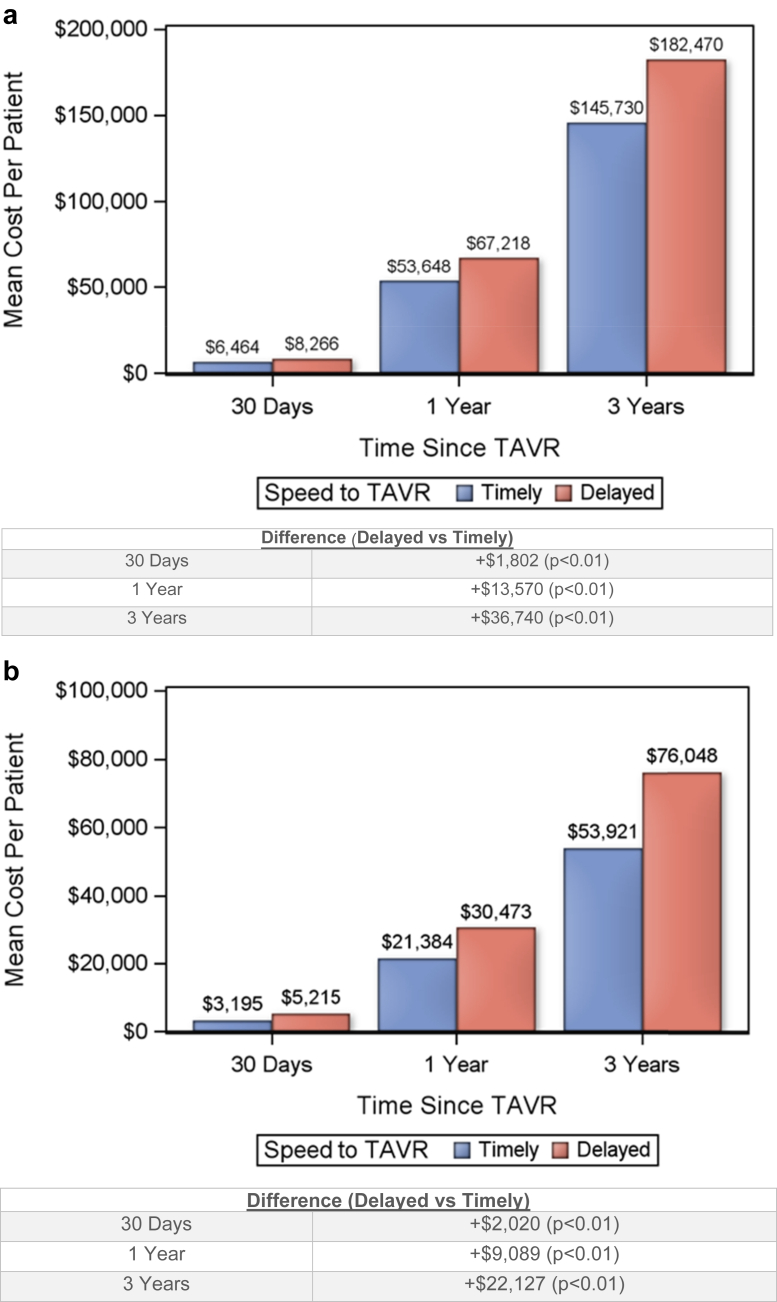
(a) Total cost of delayed versus timely TAVR at 30 days, 1 year, and 3 years. (b) Total hospitalization cost of delayed versus timely TAVR at 30 days, 1 year, and 3 years Abbreviations: TAVR, transcatheter aortic valve replacement.

Delayed TAVR was associated with substantially higher health care costs throughout the 3-year follow-up period (Figure 3). At 30 days post-TAVR, delayed patients had $1802 higher mean costs compared to timely patients ($8266 vs. $6,464; *p* < 0.01). This cost differential increased over time, reaching $13,570 at 1 year ($67,218 vs. $53,648; *p* < 0.01) and $36,740 at 3 years ($182,470 vs. $145,730; *p* < 0.01) (Figure 3A). The delayed group consistently demonstrated approximately 25% higher total health care costs after the initial 30-day period. Hospitalization costs represented the primary driver of these increased expenses (Figure 3B). At 3 years, delayed TAVR patients had $22,127 higher hospitalization costs than timely patients ($76,048 vs. $53,921; *p* < 0.01). Hospitalization costs accounted for approximately 60% of the total cost difference between groups.

### Health Care Utilization

Detailed analysis of hospitalization patterns revealed that delayed TAVR patients had consistently higher health care utilization across all categories and time periods (Table 2). The increased costs were driven by both higher hospitalization rates and more frequent readmissions.

**Table 2:** Hospitalizations by delayed versus timely TAVR at 30 days, 1 year, and 3 years

	Timely	Delayed	Difference (delayed – timely)	Incident rate ratio (delayed/timely)	
				Ratio (95% CI)	*p* value
Total hospitalizations					
30 d	0.17	0.22	0.05	1.28 (1.11, 1.47)	<0.01
1 y	0.80	1.06	0.26	1.32 (1.24, 1.41)	<0.01
3 y	2.07	2.71	0.64	1.31 (1.26, 1.36)	<0.01
Cardiac hospitalizations					
30 d	0.14	0.18	0.04	1.29 (1.10, 1.51)	<0.01
1 y	0.53	0.73	0.20	1.39 (1.29, 1.51)	<0.01
3 y	1.24	1.69	0.45	1.36 (1.29, 1.43)	<0.01
Heart failure hospitalizations					
30 d	0.05	0.09	0.04	1.68 (1.33, 2.11)	<0.01
1 y	0.25	0.45	0.20	1.82 (1.63, 2.02)	<0.01
3 y	0.59	1.04	0.45	1.76 (1.64, 1.88)	<0.01

HF, heart failure; TAVR, transcatheter aortic valve replacement.

Over the 3-year follow-up period, delayed TAVR patients experienced 2.71 total hospitalizations per patient compared to 2.07 in the timely group, representing 0.64 additional hospitalizations per patient (incidence rate ratio (IRR): 1.31; 95% CI: 1.26-1.36; *p* < 0.01). This pattern was evident as early as 30 days post-TAVR, with delayed patients having 28% higher hospitalization rates (IRR: 1.28; *p* < 0.01). Cardiac-related hospitalizations accounted for a substantial portion of this increased utilization. At 3 years, delayed patients averaged 1.69 cardiac hospitalizations compared to 1.24 in the timely group (IRR: 1.36; 95% CI: 1.29-1.43; *p* < 0.01), representing 0.45 additional cardiac admissions per patient. HF hospitalizations, a subset of cardiac-related hospitalizations showed the most pronounced differences, with delayed patients experiencing 76% higher rates at 3 years (1.04 vs. 0.59 hospitalizations per patient, IRR: 1.76; 95% CI: 1.64-1.88; *p* < 0.01). This pattern was consistent across all time points, with delayed patients showing 68% higher HF hospitalization rates even at 30 days (IRR: 1.68; *p* < 0.01).

### Sensitivity Analysis

To assess the robustness of our findings and address potential confounding from urgent/emergent cases, we performed a sensitivity analysis restricted to elective TAVR procedures only (Appendix B). This analysis included 3533 patients: 2051 timely TAVR patients (58.1%) and 1482 delayed elective TAVR patients (41.9%).

Baseline characteristics in the elective-only cohort remained well-balanced between groups, though the exclusion of urgent/emergent cases resulted in reduced differences in frailty scores. The Hospital Frailty Risk Score difference narrowed to 10.2 ± 9.2 for delayed versus 8.5 ± 7.7 for timely patients, compared to the 28% difference observed in the full cohort.

Despite removing the highest-risk delayed patients, the clinical and economic consequences of delayed TAVR persisted. The 3-year composite outcome rates remained significantly elevated in the delayed group, and as in the main analysis, all individual clinical endpoints with the exception of disabling stroke showed statistically significant differences favoring timely intervention. Economic outcomes demonstrated remarkable consistency with the primary analysis. At 3 years, delayed elective TAVR patients incurred $31,990 additional health care costs compared to timely patients, representing 87% of the cost difference observed in the full cohort. Similarly, hospitalization costs remained $18,770 higher in the delayed group, accounting for approximately 85% of the difference seen in the complete analysis.

HF hospitalizations continued to show the strongest association with delayed treatment, with a 61% increased risk (IRR: 1.61; 95% CI: 1.49-1.74; *p* < 0.01) at 3 years, only modestly attenuated from the 76% increase observed in the full cohort.

## Discussion

This comprehensive analysis of 4069 patients with clinically significant AS demonstrates that delays in TAVR beyond 90 days are associated with substantial and persistent clinical and economic consequences. Nearly half of patients experienced delayed treatment, with those receiving delayed TAVR showing an increased risk of the composite endpoint of death, HF hospitalization, or stroke at 3 years. Delayed patients had 50% higher mortality (HR: 1.50), 59% higher risk of HF hospitalizations (HR: 1.59), and a nonsignificant trend toward higher risk of disabling stroke (HR: 1.25), and incurred $36,740 in additional health care costs over the 3-year follow-up period. Importantly, these adverse outcomes persisted even when the analysis was restricted to elective procedures only, indicating that the consequences of delayed treatment extend beyond urgent or emergent cases to affect patients with discretionary procedure timing. The clinical and economic burden was evident immediately postprocedure and continued throughout the entire follow-up period.

These findings are consistent with and extend the growing body of evidence documenting the adverse consequences of delayed TAVR. Our observed 50% increase in 3-year mortality aligns with these prior reports while providing more comprehensive long-term follow-up data. The economic burden we identified builds upon recent work by Sethi and colleagues, who reported $28 in additional daily health care costs among Medicare Advantage beneficiaries with delayed TAVR translating to over $10,000 in excess costs for patients waiting 1 year for intervention.^7^ These additional costs appeared to be largely driven by non-TAVR-related health care utilization, suggesting that delays lead to progressive clinical deterioration requiring increased medical management. Our analysis extends these findings by demonstrating that the $36,740 in excess 3-year costs are primarily driven by increased hospitalizations, particularly for HF, rather than the TAVR procedure itself. The predominance of HF hospitalizations as the primary driver of both clinical events and health care costs supports previous observations that delays in symptomatic AS lead to progressive cardiac decompensation that may not fully reverse even after successful valve replacement.^13^^,^^14^ Prolonged exposure to pressure overload in AS results in left ventricular hypertrophy, myocardial fibrosis, and diastolic dysfunction that can persist despite successful valve replacement. Studies have demonstrated incomplete left ventricular mass regression and persistent myocardial fibrosis following TAVR, particularly in patients with longer duration of severe stenosis. These irreversible structural changes may explain the sustained increase in HF hospitalizations observed in our delayed treatment group, as the cardiac remodeling process may be too advanced to fully recover even after hemodynamically successful valve replacement.^15^

The mechanisms underlying these worse outcomes likely reflect the progressive nature of AS and its systemic consequences during treatment delays. Delayed patients demonstrated higher costs and worse clinical outcomes from the immediate postprocedural period through long-term follow-up and persistently elevated hospitalization rates across all time periods. Our sensitivity analysis confirmed that these adverse consequences represent genuine effects of treatment delay rather than confounding from urgent/emergent cases, suggesting that the observed associations may reflect irreversible cardiac damage and systemic deterioration that occurs during delays and persists even after successful valve replacement.

Several factors contribute to delays in TAVR access, including referral patterns, recognition of disease, patient preferences, and structural issues in the health care system.^16^ Geographic barriers represent a major challenge, with significant disparities in TAVR access across different regions, particularly with lower population densities, higher area deprivation indices, and more rural settings, where increased travel distance significantly correlates with lower TAVR utilization.^17^ Limited access to TAVR is also mediated by socioeconomic factors, with people living in high deprivation areas having less access to life-saving technologies such as TAVR compared to other widely available procedures.^18^ Medicare's reimbursement structure further compounds access issues, as regional variation in wage index adjustment levels correlates with differential TAVR utilization, with areas having lower wage indices showing substantially reduced TAVR use rates.^19^

### Limitations

Several limitations should be acknowledged. The use of administrative data and International Classification of Diseases coding to define clinically significant AS and outcomes may introduce misclassification bias, though our requirement for echocardiographic documentation helps mitigate this concern. We could not control for unmeasured confounders, including left ventricular ejection fraction, post-TAVR medical therapies (particularly sodium-glucose cotransporter 2 inhibitors and Angiotensin-Converting Enzyme (ACE) inhibitors), frailty assessments beyond administrative scores, or provider-level factors that may influence both treatment timing and outcomes. Additionally, our analysis was limited to patients within the Market Clarity database, which represents approximately 1% of annual US TAVR volume, and center-level information is not available in this deidentified database. While our cohort's baseline characteristics are generally consistent with contemporary TAVR populations, we cannot verify whether treatment patterns or unmeasured patient characteristics differ from the broader national TAVR population, which may limit generalizability. Finally and notably, our findings likely underestimate the true consequences of delayed TAVR due to survival bias. Patients in the delayed group who died prior to receiving TAVR were not included in our analysis, potentially selecting for a healthier delayed cohort. Despite this bias favoring the delayed group, we observed significantly worse outcomes, suggesting the actual impact of treatment delays may be even more pronounced than our results indicate.

## Conclusions

In this large, real-world analysis of patients with clinically significant AS, delays in TAVR beyond 90 days were associated with significantly worse clinical outcomes and substantially higher health care costs that persisted throughout 3 years of follow-up. The 50% increased mortality risk, 76% higher HF hospitalization rates, and $36,740 in excess health care costs at 3 years after TAVR associated with delayed treatment represent clinically meaningful and economically substantial consequences. These findings remained robust even when restricted to elective procedures, indicating that the adverse effects of delayed TAVR extend beyond urgent cases to affect all patients experiencing treatment delays. The immediate and persistent nature of these consequences underscores the importance of timely intervention in symptomatic AS and supports health care system investments in optimizing TAVR access and reducing wait times. As demand for TAVR continues to grow, these results provide compelling evidence for prioritizing prompt treatment to improve both patient outcomes and health care resource utilization.

## Ethics Statement

As this was a noninterventional, retrospective, observational study that collected de-identified data for patients who met eligibility criteria, informed consent was not required from patients under an institutional review board exemption status. All aspects of this study were conducted in compliance with the Health Insurance Portability and Accountability Act of 1996 regulations and the act’s Omnibus Rule of 2013.

## Funding

This study was sponsored by 10.13039/100006520Edwards Lifesciences.

## Disclosure Statement

S. Vemulapalli reports grants and/or contracts from 10.13039/100011949Abbott Vascular, the 10.13039/100005485American College of Cardiology, the 10.13039/100000050National Heart, Lung, and Blood Institute (R01HL168940-01A1, R01HL141213-03, U24HL165029-02, and U24HL171356-01A1), 10.13039/100008497Boston Scientific, 10.13039/100014941Cytokinetics, 10.13039/100006520Edwards Lifesciences, and the 10.13039/100000038Food and Drug Administration; and has served as an advisory board member, consultant, or speaker for 10.13039/100011949Abbott Vascular, the 10.13039/100009965American College of Physicians, 10.13039/100004325AstraZeneca, 10.13039/100001003Boehringer Ingelheim, 10.13039/100014941Cytokinetics, 10.13039/100006520Edwards Lifesciences, 10.13039/100020588HeartFlow, 10.13039/100019998JenaValve, Ikon, 10.13039/100004374Medtronic, Medscape, and Total CME. M. Russo received research grants from 10.13039/100006520Edwards Lifesciences, 10.13039/100019998JenaValve, and 10.13039/100000046Abbott; and has served as a consultant or advisor for 10.13039/100000046Abbott and 10.13039/100006520Edwards Lifesciences. S. Murphy, S. Chikermane, and S. Clancy are all employees of 10.13039/100006520Edwards Lifesciences. C. Stinis is a consultant for and receives honorarium from 10.13039/100006520Edwards Lifesciences, 10.13039/100004374Medtronic, Shockwave Medical, and 10.13039/100008497Boston Scientific.
