# Supplementary material for: The Clinical and Economic Consequences of Delayed Transcatheter Aortic Valve Replacement
Source: Struct Heart. 2025 Oct 25;10(5):100742. doi: 10.1016/j.shj.2025.100742 (PMC13250312; doi:10.1016/j.shj.2025.100742)
Supplement: Appendix [file mmc1.docx]

**Appendix A TAVR by Year**

| **TAVR Year** | **Timely** | **Delayed** | **Overall** |
| --- | --- | --- | --- |
| **2019** | 248 (45.8%) | 294 (54.2%) | 542 (100%) |
| **2020** | 494 (49.4%) | 505 (50.6%) | 999 (100%) |
| **2021** | 532 (50.7%) | 518 (49.3%) | 1050 (100%) |
| **2022** | 537 (54.5%) | 449 (45.5%) | 986 (100%) |
| **2023** | 240 (48.8%) | 252 (51.2%) | 492 (100%) |
| **Total** | 2051 | 2018 | 4069 |

**Appendix B Sensitivity Analysis Limited to Elective TAVRs**

**Table A1 Patient Characteristics at Time of Clinically Significant AS Diagnosis for Elective TAVRs**

| Model | | Timely TAVR | Delayed TAVR | Total |
| --- | --- | --- | --- | --- |
| Sample Size | | 2,051 (58.1% of Total) | 1,482 (41.9% of Total) | 3,533 (100%) |
| Alive and enrolled in follow-up period after TAVR discharge | | 2,046 (99.8%) | 1,473 (99.4%) | 3,519 (99.6%) |
| Age (in years) | | 76.5 (7.9) | 75.7 (8.1) | 76.2 (8) |
| Female | | 872 (42.5%) | 609 (41.1%) | 1,481 (41.9%) |
| Caucasian Race/Ethnicity | | 1,886 (92%) | 1,346 (90.8%) | 3,232 (91.5%) |
| Payor | Commercial | 315 (15.4%) | 224 (15.1%) | 539 (15.3%) |
|  | Medicare | 1,699 (82.8%) | 1,226 (82.7%) | 2,925 (82.8%) |
|  | Other | 37 (1.8%) | 32 (2.2%) | 69 (2%) |
| Region | Midwest | 871 (42.5%) | 709 (47.8%) | 1,580 (44.7%) |
|  | Northeast | 379 (18.5%) | 306 (20.6%) | 685 (19.4%) |
|  | South | 528 (25.7%) | 283 (19.1%) | 811 (23%) |
|  | West | 273 (13.3%) | 184 (12.4%) | 457 (12.9%) |
| AVR Year | 2019 (July – Dec) | 248 (12.1%) | 212 (14.3%) | 460 (13%) |
|  | 2020 | 494 (24.1%) | 377 (25.4%) | 871 (24.7%) |
|  | 2021 | 532 (25.9%) | 369 (24.9%) | 901 (25.5%) |
|  | 2022 | 537 (26.2%) | 333 (22.5%) | 870 (24.6%) |
|  | 2023 (Jan – Jun) | 240 (11.7%) | 191 (12.9%) | 431 (12.2%) |
| Elixhauser Score | | 6.6 (2.7) | 6.4 (2.8) | 6.5 (2.7) |
| Hospital Frailty Risk Score | | 8.5 (7.7) | 10.2 (9.2) | 9.3 (8.4) |
| Bicuspid Aortic Valve | | 108 (5.3%) | 58 (3.9%) | 166 (4.7%) |
| Urgent/Emergent TAVR* | | 0 (0%) | 0 (0%) | 0 (0%) |

**Figure A2 Adjusted Risk of Composite Endpoint (Death, Disabling Stroke or Heart Failure Readmission) and each Component at 3 years for Elective TAVRs**

**
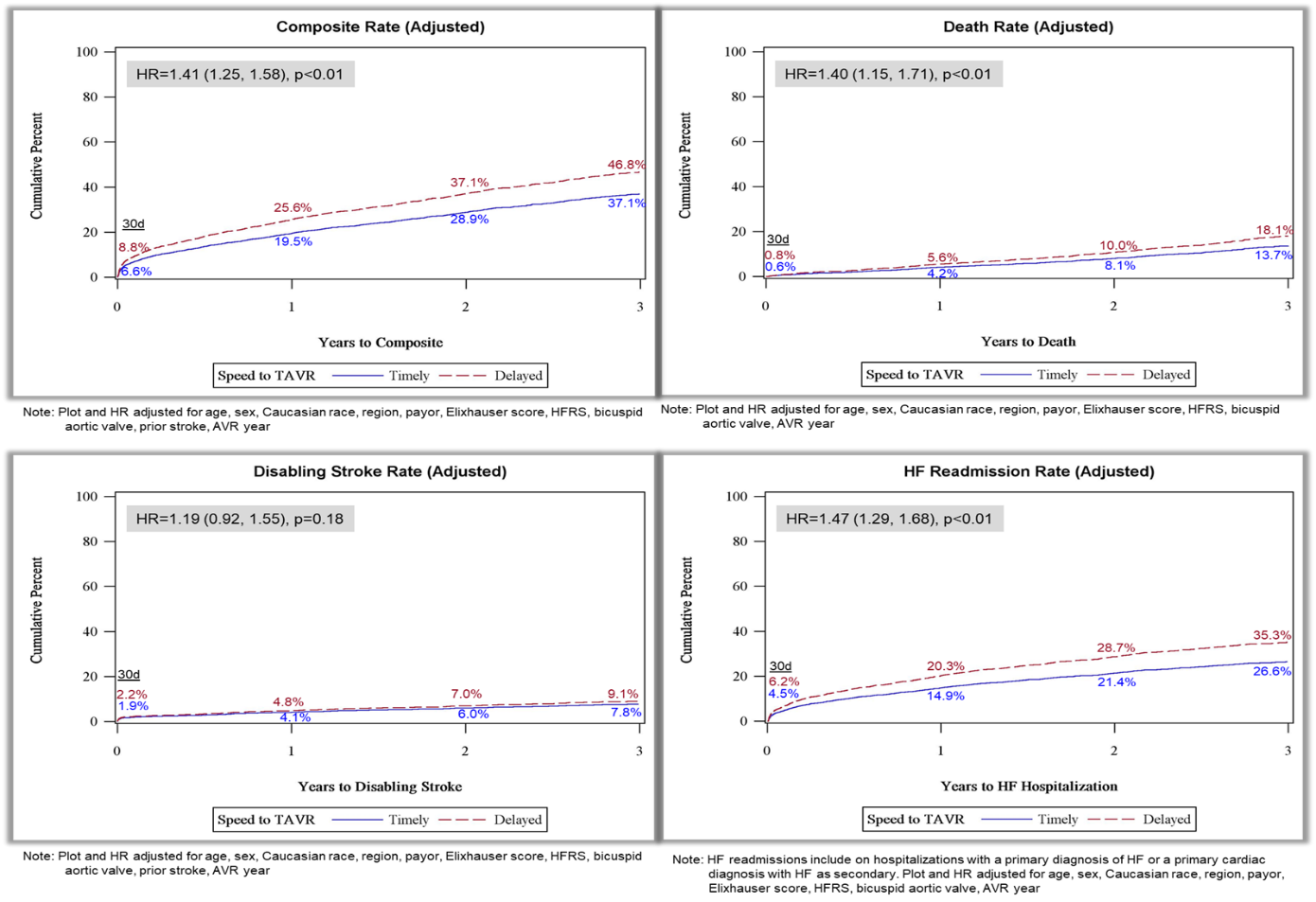
**

**Figure A3A Total Cost of Delayed versus Timely TAVR at 30 Days, 1 Year and 3 Years for Elective TAVRs**

**
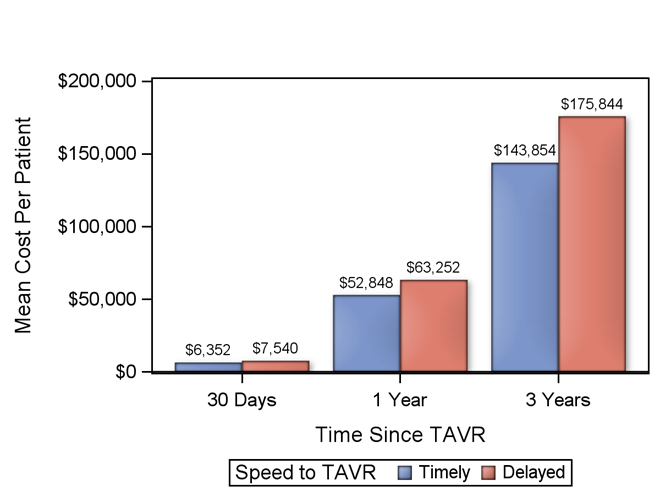
**

| Difference (Delayed vs Timely) | |
| --- | --- |
| 30 Days | +$1,188 (p<0.01) |
| 1 Year | +$10,404 (p<0.01) |
| 3 Years | +$31,990 (p<0.01) |

**Figure A3B Total Hospitalization Cost of Delayed versus Timely TAVR at 30 Days, 1 Year and 3 Years for Elective TAVRs**

**
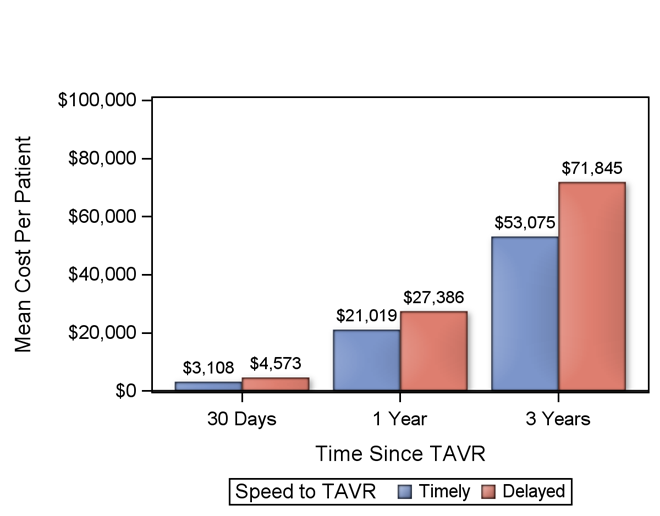
**

| Difference (Delayed vs Timely) | |
| --- | --- |
| 30 Days | +$1,465 (p<0.01) |
| 1 Year | +$6,367 (p<0.01) |
| 3 Years | +$18,770 (p<0.01) |

**Table A2 Hospitalizations by Delayed versus Timely TAVR at 30 Days, 1 Year and 3 Years for Elective TAVRs**

|  | Timely | Delayed | Difference  (Delayed – Timely) | Incident Rate Ratio  (Delayed / Timely) | |  |
| --- | --- | --- | --- | --- | --- | --- |
|  |  |  |  | Ratio (95% CI) | P-Value |  |
| **Total Hospitalizations** | | | | | | |
| 30 Days | 0.17 | 0.21 | 0.04 | 1.21 (1.03, 1.41) | 0.02 |  |
| 1 Year | 0.79 | 1.00 | 0.21 | 1.27 (1.18, 1.36) | <.01 |  |
| 3 Years | 2.03 | 2.62 | 0.59 | 1.29 (1.24, 1.35) | <.01 |  |
| **Cardiac Hospitalizations** | | | | | | |
| 30 Days | 0.14 | 0.17 | 0.03 | 1.22 (1.02, 1.44) | 0.03 |  |
| 1 Year | 0.52 | 0.68 | 0.16 | 1.31 (1.21, 1.43) | <.01 |  |
| 3 Years | 1.22 | 1.60 | 0.38 | 1.31 (1.24, 1.38) | <.01 |  |
| **Heart Failure Hospitalizations** | | | | | | |
| 30 Days | 0.05 | 0.07 | 0.02 | 1.38 (1.06, 1.79) | 0.02 |  |
| 1 Year | 0.24 | 0.39 | 0.15 | 1.60 (1.42, 1.80) | <.01 |  |
| 3 Years | 0.58 | 0.94 | 0.36 | 1.61 (1.49, 1.74) | <.01 |  |
